# Study of the reappearance of sieve plate-like pores in immortalized sinusoidal endothelial cells – Effect of actin inhibitor in mixed perfusion cultures

**DOI:** 10.1186/1476-5926-2-S1-S28

**Published:** 2004-01-14

**Authors:** Masaya Saito, Tomokazu Matsuura, Takahiro Masaki, Haruka Maehashi, Filip Braet

**Affiliations:** 1 Division of Gastroenterology and Hepatology, Department of Internal Medicine, The Jikei University School of Medicine Tokyo, Japan; 2 Department of Laboratory Medicine, Jikei University School of Medicine, Japan; 3 Kanagawa Prefectural Cancer Center Hospital, Japan; 4 Department of Biochemistry, The Jikei University School of Medicine, Japan; 5 Laboratory for Cell Biology and Histology, Free University of Brussels, Belgium; 6 Present address: Department for Molecular Biomedical Research, Molecular Cell Biology Unit, Ghent University (UGhent), Technologiepark 927, 9052 Zwijnaarde, Belgium

## Introduction

We previously reported that when the high-functioning human hepatoma cell line, FLC-5, immortalized sinusoidal endothelial cell line, M1, and immortalized hepatic stellate cell line, A7, were cultured in the 3-dimensional filled type bioreactor, tissue reorganization resembling that seen in the live liver occurred, with the appearance of pores in the sinusoidal endothelial cells (SECs) [1]. The process and mechanism of formation of these pores remain unclarified. The presence of actin at the margin of these pores has been demonstrated by electron microscopic study [2]. Swinholide-A, which is actin inhibitor synthesized from Okinawa sponge, increase the number of pores on primary culture on SECs derived from the rat [3]. In present study, we examine whether or not the pores on SECs under three-dimensional perfusion co-culture treatment with Swinholide-A behave like those in primary culture cells.

## Methods

2 – 10^7 ^FLC-5 cells were inoculated into the reservoir, and they were perfused slowly in bioreactor for 2 hours in closing circuit to induce the cells to adhere to the porous cellulose beads (Asahi Kasei Co., Ltd.) that served as the carrier. Five days later, 2 – 10^7 ^A7 cells were added in a similar manner, followed another 5 days later by the addition of the M1 cells. After perfusion for 1 hour with medium supplemented with 100 nM Swinholide-A, or for 2 hours with the medium supplemented with 200 nM Swinholide-A, the cellulose beads along with the adherent cells were withdrawn from bioreactor.

For scanning electron microscopy (SEM), cultured cells ware fixed 1.2% glutaraldehyde in 0.1 M phosphate buffer (PB), pH7.4 and postfixed with 1% OsO_4 _in 0.1 M PB. The fixed cells were rinsed twice with PBS, subsequently dehydrated in ascending concentration of ethanol, critical point-dried using carbon dioxide and coated by vacuum evaporated carbon and ion-spattered gold. Specimens were observed by JSM-35 (JEOL, Tokyo) at an accelerated voltage of 10 kV. For transmission electron microscopy (TEM), cultured cells were fixed with 2.0% glutaraldehyde in 0.1 M PB and postfixed with 1% OsO_4 _in 0.1 M PB. Specimens were dehydrated in ethanol, and embedded in mixture of Epon-Araldite. Thin sections were made with a diamond knife mounted on a LKB ultratome, and stained with aqueous uranyl acetate. Specimens were examined with a JEOL 1200EX electron microscopy.

## Results

**Figure 1:**
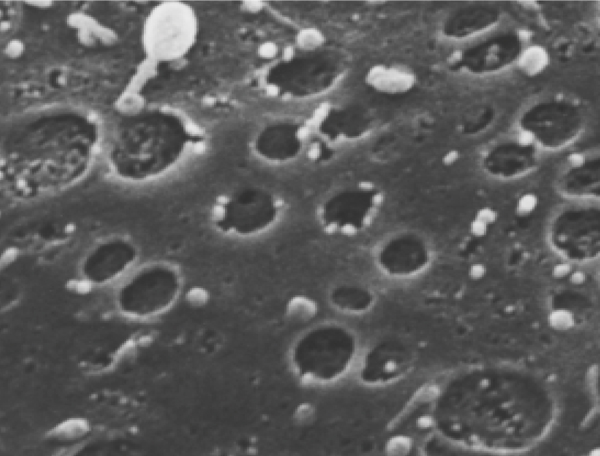
Scanning electron microscopy views of M1 cell. Large pores can be observed on surface of M1 cell. Small pores are noted in the large pores.

When M1 cells were incubated in plastic dish or independently in radial-flow bioreactor filled with glass beads [4], no pores were observed. However, pits with a diameter of nanometers were visible in both cases. FLC5, A7, and M1 were co-cultured in radial-flow bioreactors, and M1 cells grew to cover the entire surface of the perfused side of culture medium. Under SEM, the cells treated with Swinholide-A (100 nM) for 1 hour showed an increased number of pores of 100-nm diameter and with 200 nM for 2 hour had an even larger number of pores and some of them had a large number of the larger pits about 1 micrometer and depressions (Fig. 1).

**Figure 2:**
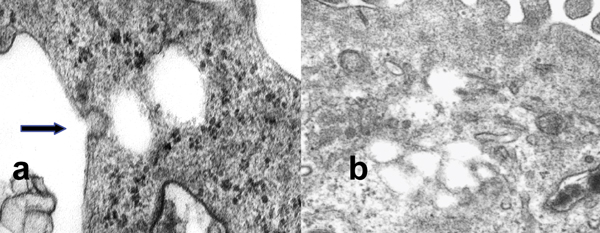
Transmission electron microscopic of M1 cell. a: Invagination of plasma membrane like caveolae (arrow) and fenestrae like pores can be observed. b: The pores are fused and interconnected labyrinth-like structure.

Small pores were abundant in the depressed area. Under TEM, vacuoles, coincident with vesiculo-vacuolar organelles (VVOs) about 200 nm in size, were noted. Junctional complex were observed between M1 and A7, M1 and M1. Invaginations of plasma membrane like caveola can be observed (Fig. 2a). We could also observe that caveola was fused and made the interconnected labyrinth-like structure (Fig. 2b).

## Discussion

The number of pores on M1 cells in co-culture using RFB was increased by the effect of actin inhibitor. It is possible that the mechanism of formation of pores can be investigated by using RFB with actin inhibitor. Several authors reported that share stress alternated the cellular cytoskeleton such as actin. Nevertheless, we could not observe the fenestrae on M1 cells that was monocultured by RFB [4]. It is suggested that both of share stress and cell-to-cell interaction are important factors of reappearance of fenestrae in immortalized endothelial cell. The 3-dimensional perfusion co-culture using RFB is useful for reconstruction of liver tissue *in vitro *and increasing responsibility of drug.
